# Cucurbitacin-B Exerts Anticancer Effects through Instigation of Apoptosis and Cell Cycle Arrest within Human Prostate Cancer PC3 Cells via Downregulating JAK/STAT Signaling Cascade

**DOI:** 10.3390/ph15101229

**Published:** 2022-10-06

**Authors:** Ahmed Alafnan, Abdulwahab Alamri, Talib Hussain, Syed Mohd Danish Rizvi

**Affiliations:** 1 Department of Pharmacology and Toxicology, College of Pharmacy, University of Ha’il, Ha’il 81442, Saudi Arabia; 2 Department of Pharmaceutics, College of Pharmacy, University of Ha’il, Ha’il 81442, Saudi Arabia

**Keywords:** prostate cancer, Cucurbitacin-B, apoptosis, notch signaling, cell cycle arrest

## Abstract

Cucurbitacin-B (Cur-B) is an analogue triterpenoid belonging to the Cucurbitaceae family. Previous reports have explicitly outlined various biological activities of Cucurbitaceae family members, including the anticancer activity of Cur-B. In the present study, we tried to elucidate the anticancer efficacy of Cur-B against prostate cancer PC3 cells. PC3 cells were exposed to purified Cur-B at 5, 10, 15, 20 and 25 µM for 24. Cur-B exposure reduced cell viability of PC3 cells at 5 µM (*p* < 0.05), with further reduction with increased Cur-B concentration (15 µM, *p* < 0.01 and 25 µM, *p* < 0.001). Cur-B also succeeded in instigating nuclear fragmentation and condensation, followed by activation of caspase-8, -9 and -3 proportionally with increasing concentrations of Cur-B. Treatment with Cur-B also instigated ROS-mediated oxidative stress both qualitatively and quantitatively at 5 µM, *p* < 0.05; 15 µM, *p* < 0.01 and 25 µM, *p* < 0.001. Increased ROS after Cur-B treatment also led to dissipation of mitochondrial membrane potential, thereby resulting in considerable apoptosis (*p* < 0.001), which, again, was proportionally dependent on Cur-B concentration. Cur-B exposure to PC3 cells was concomitantly followed by reduced cyclin D1, cyclin-dependent kinase 4 (CDK4) expression and augmented mRNA expression of CDK inhibitor p21^Cip1^. Intriguingly, Cur-B exposure also led to considerable downregulation of the JAK/STAT signaling cascade, which may be the reason behind Cur-B-mediated apoptosis and cell cycle arrest within PC3 cells. Therefore, these observations explicitly establish that Cur-B could serve in the prevention of prostate cancer.

## 1. Introduction

Prostate cancer (PCa) is a serious malignancy among men globally and was positively diagnosed in 1,414,259 men globally during 2020, which constituted 7.3% of the total 19,292,789 cases of cancer. Furthermore, 375,304 causalities due to prostate cancer were reported globally in the year 2020 [1]. The disease is quite frequently reported in males during the sixth and/or seventh decade of their life, which, in turn, results in increased incidence of PCa-related mortality and morbidity. Although complete insight into the cellular and molecular events leading to the onset and progression of PCa are yet to be explored fully, several risk factors have been associated with the disease. Among these risk factors, important ones include androgen metabolism, diet, ethnicity and the mutations in oncogenes. The onset of PCa is related to subtle genetic mutations either in nuclear or cytoplasmic constituents of the cells [2].

JAK/STAT signaling is initiated after JAK activation through its ligand and includes interferons and growth factors for particular transmembrane receptors [3]. Among members of this family, JAK2 is associated with regulating expression of downstream genes such as signal transducers and activators of transcription (STATs), which subsequently modulate the expression of genes responsible for instigation and/or amelioration of apoptosis. Undoubtedly, JAK/STAT signaling is a prerequisite for prolonged survival and proliferation of various cancer cells and may also be implicated with resistance of cancer cells against different chemotherapeutics [4]. Another important family of transcription factors is the signal transducers and activators of transcription (STATs), which are closely associated with the onset and progression of various carcinomas, including PCa. STATs were also recently implicated with resistance against chemotherapeutics, namely, chemotherapy and anti-androgens in PCa. These factors are further reported to elevate metastasis in PCa and are commonly upregulated during advanced grade PCa [5].

Recent advancements in technologies and an in-depth understanding of PCa pathogenesis has led to the elucidation of several important therapeutical interventions for this pernicious disease, including surgical and/or radiological interventions and ablation of androgens. However, the efficacy of these treatment modalities is dependent upon the stage at which PCa is diagnosed, thereby necessitating the surge for exploration of options that can aid in amelioration of PCa independent of the stage at which it is diagnosed. Intriguingly, the present standard clinical management of the disease relies upon the use of docetaxel, which is reported to exert its effect by destabilizing the microtubules [6]. However, docetaxel has also been previously shown to instigate resistance due to mutations within microtubules and simultaneous efflux of the drug [7,8]. Additionally, another major limitation in the use of standard chemotherapeutics are their associated side-effects, which, catastrophically, have a negative impact on hair follicles, blood cells and cells of the digestive tract and mouth [8].

Among different known vegetable and fruit crops, *Cucurbitaceae* represents the largest family, constituting nearly 125 genera along with 960 species. The members of the *Cucurbitaceae* family have been known to be a part of ancient culinary and medical traditions. Intriguingly, they are also an integral part of folk medicines and have previously been mentioned in Ayurveda for medicinal benefits [9]. Several members of the *Cucurbitaceae* family play beneficial roles in different ailments. Chemically, cucurbitacins are recognized as a triterpenoid constituted by a nuclear skeleton of a 19-(10”9β)-abeo-5α-lanostane base that carries oxygen atoms at varying positions [10]. Recently, the anticancer efficacy of cucurbitacin and its derivatives was documented against human breast cancer MCF-7 and MDA-MB-231 cells [11]. However, mechanistic insight into the anticancer efficacy of Cur-B and its plausible role in modulating JAK/STAT signaling cascade remains unexplored in androgen-independent human prostate cancer PC-3 cells. Thus, we hypothesized that Cur-B mediates anti-cancer effects against human prostate cancer PC-3 cells via modulation of JAK/STAT signaling components.

## 2. Results

### 2.1. Cur-B Induced Significant Cytotoxic Effects on PC3 Cells

**Figure 1:**
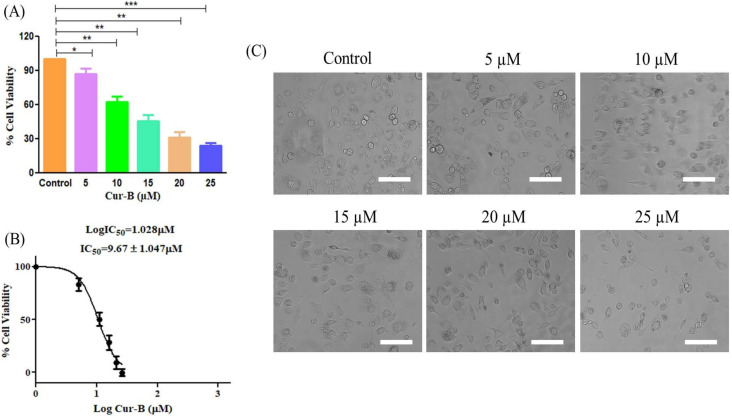
Cytotoxic effects of Cur-B against PC-3 cells as evaluated through MTT assay: (**A**) changes in cell viability percentage, (**B**) IC_50_ value of Cur-B against PC-3 cells and (**C**) changes in morphology of PC-3 cells upon exposure to various Cur-B concentrations after 24 h. Scale bar = 100 µm. * *p* < 0.05, ** *p* < 0.01, *** *p* < 0.001.

The cytotoxicity of Cur-B against PC3 cells was estimated using tetrazolium-based MTT assay. During the assay, it was observed that Cur-B significantly reduced the cell viability of PC3 cells to 87.13% ± 3.81% (5 µM), 62.42% ± 3.76% (10 µM), 45.73% ± 4.25% (15 µM), 31.26% ± 3.71% (20 µM) and 24.25% ± 1.95% (25 µM) in comparison with the untreated control (Figure 1A). Intriguingly, the IC50 value of Cur-B was calculated to be 9.67 ± 1.04 µM within PC-3 cells (Figure 1B).

### 2.2. Cur-B Exposure Altered PC3 Morphology

Morphological assessment of Cur-B-treated PC3 cells was done using a microscope, and it was observed that Cur-B instigated morphological alterations within PC3 cells after 24 h of exposure. The photomicrograph as shown in Figure 1C clearly shows that PC3 cells obtained a circular and slightly shrunken morphology with increased concentrations of Cur-B. A considerable fraction of PC3 cells also exhibited cell membrane lysis, swelling and organelle disintegration. However, the untreated control PC3 cells showed normal/flattened morphology and were well-spread. Thus, it became evident that Cur-B exerted cytotoxic effects against PC3 cells proportional to its concentration.

### 2.3. Cur-B Instigated Nuclear Condensation

**Figure 2:**
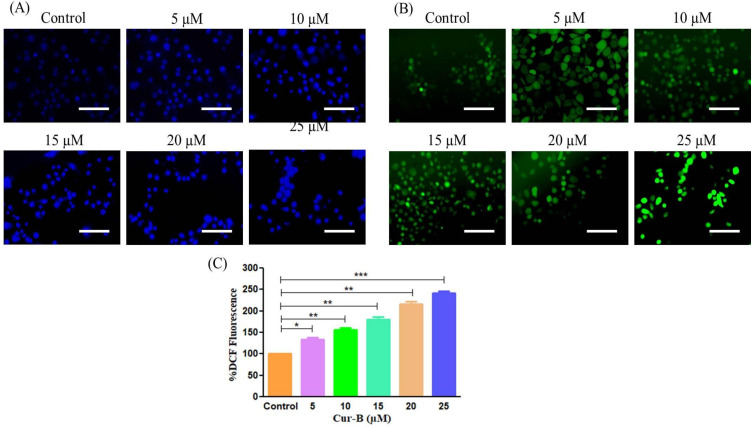
The efficacy of Cur-B in instigating (**A**) nuclear condensation and (**B**) intracellular ROS production, and (**C**) quantification of ROS production in PC-3 cells. Scale bar = 100 µm. * *p* < 0.05, ** *p* < 0.01, *** *p* < 0.001.

Nuclear fragmentation and condensation are important indicators of alteration within the nucleus during the onset of apoptosis. Hoechst-33342 dye was used to qualitatively assess whether Cur-B-mediated cytotoxicity in PC3 cells was due to the instigation of apoptotic cell death. Significant alterations within nuclear morphology were seen in PC3 after 24 h of Cur-B exposure. The fluorescent photomicrographs as shown in Figure 2A revealed that Cur-B exposure resulted in increased nuclear fragmentation and condensation in PC3 cells proportional to its concentration. However, untreated control PC3 cells were devoid of any prominent blue fluorescence, indicating the absence of nuclear fragmentation and condensation.

### 2.4. Cur-B Instigated Intracellular ROS Production

Increased ROS production is associated with activation of caspase-dependent and -independent pathways resulting in apoptosis [12]. Thus, the effects of Cur-B in instigating ROS within PC3 cells was evaluated using DCF-DA stain. As shown in Figure 2B, the photomicrograph explicitly indicates increased DCFH-DA-mediated green fluorescence in PC3 cells treated with 5–25 µM Cur-B. Furthermore, quantification of DCFH-DA-mediated mean fluorescence intensity (MFI) also reaffirmed that ROS production was instigated within PC3 cells post-exposure with 5 µM Cur-B by approximately 133.71 ± 3.66% compared to the untreated control. Intriguingly, this augmentation of ROS was found to follow a dose-dependent trend, since its intracellular accumulation further escalated to 156.68 ± 3.13% (10 µM), 180.75 ± 4.07% (15 µM), 216.88 ± 4.06% (20 µM) and 241.53 ± 3.35% (25 µM), as shown in Figure 2C.

### 2.5. Cur-B Treatment Induced Dissipation of ΔΨm

**Figure 3:**
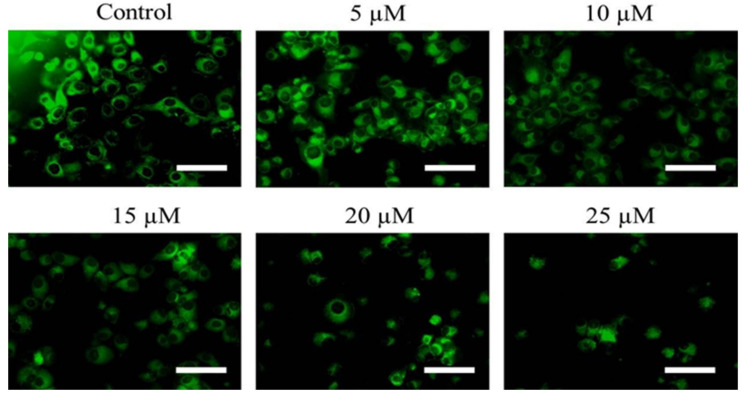
Dissipation of mitochondrial membrane potential (ΔΨm) in PC-3 cells post treatment with various Cur-B concentration as assessed through Rh123 stain. Scale bar = 100 µm.

Activation of mitochondria-dependent cell death is characterized by the onset of dissipating ΔΨm. In order to assess the efficacy of Cur-B in dissipating ΔΨm, mitochondrial potential-specific Rh-123 dye was used. The fluorescent photomicrographs shown in Figure 3 evidently showed that Cur-B exposure reduced the ΔΨm of PC3 cells in a dose-dependent manner.

### 2.6. Cur-B Instigated Apoptosis Post Exposure in PC3 Cells

**Figure 4:**
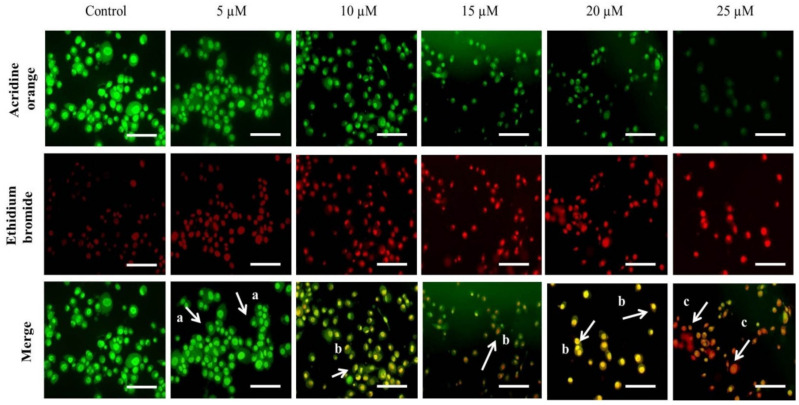
Instigation of apoptosis in PC-3 cells post treatment with stated Cur-B concentration as assessed through AO/EtBr dual staining. Scale bar = 100 µm; a, b and c represent viable, early apoptotic and late apoptotic PC-3 cells, respectively.

Furthermore, Cur-B-instigated apoptosis based on morphological attributes of the nucleus in PC-3 cells was assessed using AO/EtBr staining. AO stain is sensitive towards both live and apoptotic cells; however, EtBr is sensitive for cells with altered membrane integrity. As per the merged fluorescent photomicrograph of AO/EtBr shown in Figure 4, the live cells were stained with AO and exhibited bright green fluorescence. Intriguingly, yellow, reddish or orange indicated early and late apoptotic PC-3 cells, respectively, post-Cur-B treatment, and exhibited a dose-dependent increase in the levels of orange fluorescence. Contrastingly, PC-3 cells not exposed to 5–25 µM Cur-B exhibited bright green fluorescence, indicating the presence of live PC-3 cells.

**Figure 5:**
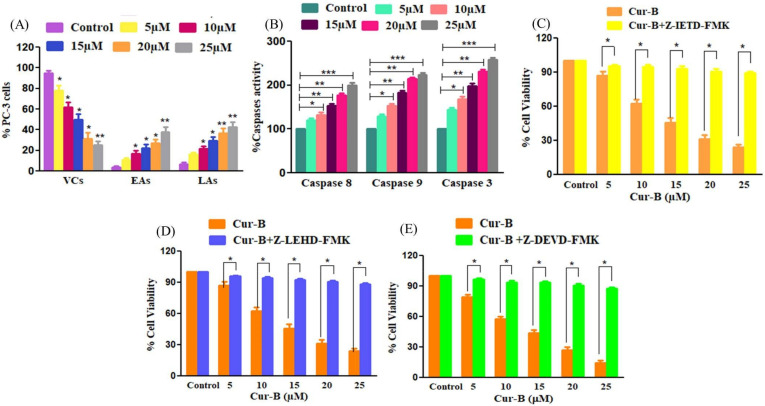
Quantification of Cur-B-instigated (**A**) apoptosis, (**B**) Cur-B mediated activation of caspase-8, -9 and -3 and (**C**–**E**) cytotoxic effects of Cur-B against caspase inhibitor pretreated PC-3 cells. VCs, viable cells; LAs, late apoptotic cells; EAs early apoptotic cells. * *p* < 0.05, ** *p* < 0.01, *** *p* < 0.001.

The quantification of early and late apoptotic cells indicated that Cur-B-mediated apoptosis accounted for 10.8% ± 1.56% of early apoptotic (EA) cells and 16.23% ± 1.46% of late apoptotic (LA) cells at 5 µM. The percentages of these EA and LA cells increased proportionally with elevated Cur-B concentration. The EA cell % was found to be 16.97% ± 2.21% (10 µM); 22.31% ± 2.76% (15 µM); 27.35% ± 2.96% (20 µM) and 37.81% ± 3.88% at 25 µM Cur-B exposure. Intriguingly, the LA cell % was subsequently found to be 21.43% ± 2.16%; 29.57% ± 3.10%; 36.79% ± 3.83% and 42.84% ± 3.74% at the stated doses of Cur-B, respectively (Figure 5A). These observations indicated that Cur-B considerably instigated apoptosis in PC-3 cells.

### 2.7. Cur-B Mediated the Activation of Caspase-8, -9 and -3

Caspases are regarded as important mediators of apoptotic cell death and are involved in proteolytic degradation of different cellular proteins. Caspases such as caspase-8 and caspase-9 are involved in triggering the extrinsic and intrinsic apoptotic pathways, respectively, whereas caspase-3 is an important downstream effector caspase. Owing to their role in instigating apoptosis, the changes in activities of different caspases was investigated. As shown in Figure 5B, significant augmentation in activities of different caspases was recorded post-24 h of exposure with varying concentrations of Cur-B. Caspase-3 activity was significantly increased by 144.69% ± 2.72% (5 µM), 168.12% ± 4.97% (10 µM), 198.87% ± 4.59% (15 µM), 231% ± 3.39% (20 µM) and 258.46% ± 3.91% (25 µM) in comparison with the untreated control PC3 cells. Moreover, caspase-8 and capsase-9 activities escalated by 119.87% ± 3.45% (5 µM), 132.43% ± 4.19% (10 µM), 152.72% ± 3.53% (15 µM), 177.31% ± 3.57% (20 µM) and 199.26% ± 4.96% (25 µM) and 129.65% ± 3.54% (5 µM), 153.73% ± 3.40% (10 µM), 183.58% ± 3.45% (15 µM), 214.51% ±2.36% (20 µM) and 223.21% ± 3.64% (25 µM) in comparison with the untreated control. These observations implicated that both intrinsic and extrinsic apoptotic pathways were involved in Cur-B-instigated apoptosis in PC3 cells.

### 2.8. Caspase Inhibitors Mitigated Cur-B-Induced Cytotoxicity

To further ascertain the involvement of caspases in Cur-B-instigated apoptotic cell death, we re-performed cell viability assays with PC3 cells pretreated with specific caspase inhibitors. As reported in Figure 5C–E, the cytotoxic effects of Cur-B were substantially reduced in cells pretreated with specific caspase inhibitors. From these observations, it became quite evident that caspase activation played a major role in inducing Cur-B-mediated apoptosis in PC3 cells. However, since the caspase inhibitors failed to completely ameliorate Cur-B-mediated apoptosis, there may be the involvement of caspase-independent pathways of apoptotic cell death.

### 2.9. Cur-B Enhances Apoptosis by Reducing Expression of Anti-Apoptotic Markers

**Figure 6:**
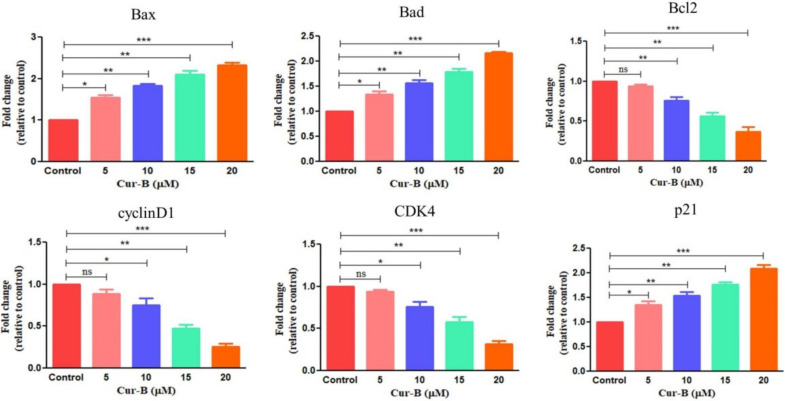
Effect of Cur-B on modulating mRNA expression of various genes involved in apoptosis and cell cycle regulation in PC-3 cells. * *p* < 0.05, ** *p* < 0.01, *** *p* < 0.001.

The expression of anti-apoptotic gene mRNA, namely Bcl-2, Bad and Bax, was estimated using ^ΔΔ^CT method and was expressed as fold change in comparison with the untreated control. The findings of the study explicitly indicated that Bcl2 mRNA expression was considerably deflated in Cur-B-treated cells with concomitant increase in the mRNA expression of Bax and Bad genes (pro-apoptotic genes). Bcl2 mRNA expression was curtailed to 0.93 ± 0.02 (5 µM); 0.75 ± 0.03 (10 µM); 0.64 ± 0.03 (15 µM) and 0.36 ± 0.05 (20 µM). However, Bad mRNA expression escalated to 1.34 ± 0.05 (5 µM); 1.56 ± 0.05 (10 µM); 1.79 ± 0.04 (15 µM) and 2.16 ± 0.02 (20 µM), whereas Bax mRNA expression escalated to 1.55 ± 0.04 (5 µM); 1.83 ± 0.03 (10 µM); 2.1 ± 0.07 (15 µM) and 2.33 ± 0.05 (20 µM) after 24 h of exposure to Cur-B (Figure 6).

### 2.10. Cur-B Modulates cyclinD1, CDK4 and p21Cip1 mRNA Expression

The effect of Cur-B on modulating expression of genes involved in the cell cycle was further evaluated through qRT-PCR. As presented in Figure 6, Cur-B reduced mRNA expression of cyclin D1, exhibiting a dose-dependent trend. Exposure of PC-3 cells to Cur-B considerably reduced expression of cyclin D1 mRNA 0.88 ± 0.04 (5 µM); 0.75 ± 0.06 (10 µM); 0.47 ± 0.03 (15 µM) and 0.25 ± 0.05 (20 µM) fold compared with untreated control. Additionally, the expression of p21Cip1 mRNA was elevated by 1.34 ± 0.06 (5 µM); 1.54 ± 0.05 (10 µM); 1.76 ± 0.03 (15 µM) and 2.09 ± 0.05 (20 µM) fold against the control. Thereafter, the effect of Cur-B in regulating CDK4 expression in PC-3 cells was further evaluated using qRT-PCR. The expression of CDK4 was curtailed up to 0.93 ± 0.02 (5 µM); 0.76 ± 0.04 (10 µM); 0.57 ± 0.05 (15 µM) and 0.31 ± 0.03 (20 µM) fold in juxtaposition with untreated control.

### 2.11. Cur-B Impeded the Expression of JAK1/STAT1 Signaling

**Figure 7:**
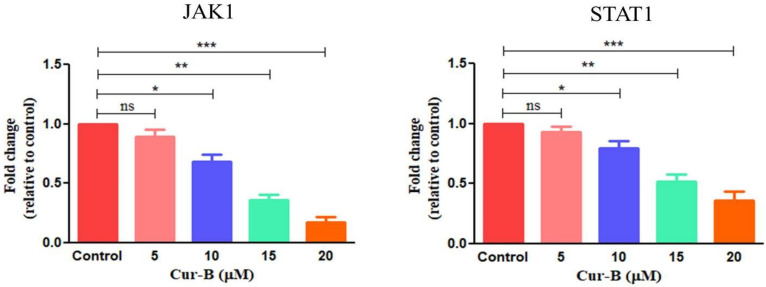
Effect of Cur-B in modulating the mRNA expression of important genes involved in JAK1/STAT1 signaling. * *p* < 0.05, ** *p* < 0.01, *** *p* < 0.001.

To understand the effect of Cur-B on JAK/STAT signaling in PC-3 cells, qRT-PCR analysis was undertaken. JAK/STAT signaling is associated with impeding apoptosis and enhancing cellular proliferation of cancer cells. Owing to this, JAK/STAT signaling is considered a putative target for the development of chemotherapeutics. Therefore, we investigated whether Cur-B showed its competence in modulating the JAK/STAT signaling cascade. We explored the effects of Cur-B exposure on key constituents of this pathway, which included JAK1 and STAT1 in PC-3 cells. As evident from Figure 7, Cur-B reduced JAK1 mRNA expression in PC-3 cells proportionally with increases in its concentration. Cur-B (5–20 µM) decreased the mRNA levels of JAK1 to 0.89 ± 0.02; 0.68 ± 0.04; 0.36 ± 0.03 and 0.17 ± 0.03 fold at respective concentration in juxtaposition with cells not exposed to Cur-B. Subsequently, the Cur-B-mediated effect on Jagged1 was further explored. It was evident that Cur-B ameliorated the mRNA expression of STAT1 to 0.93 ± 0.03 (5 µM); 0.79 ± 0.04 (10 µM); 0.52 ± 0.04 (15 µM) and 0.36 ± 0.05 (20 µM) fold in juxtaposition with cells not exposed to Cur-B (Figure 7).

## 3. Discussion

Due to the limitations of effective therapeutic strategy and the relapse of current treatment regimes, prostate cancer remains a global cause of mortality among males [13]. The last several years have witnessed a steep incline in investigative studies focusing on deciphering the anti-cancer potential of dietary substances for their antagonistic effects against angiogenesis, metastasis and tumor growth. These explorations are further strengthened by the notion that natural products exhibit relatively fewer side effects in comparison with their synthetic counterparts and can therefore be exploited for their intrinsic cancer-preventing abilities [14,15,16,17,18,19]. Cucurbitacin-B is a tetracyclic triterpenoid derived naturally from plants belonging to the Cucurbitaceae family and which previously has been reported to have anticancer effects against human osteosarcoma alone and in combinatorial approach with methotrexate [20,21]. Subsequently, Cucurbitacin-B has also been reviewed exhaustively for its anticancer effects against different carcinomas [22,23]. Thus, we tried to explore the mechanistic insight into the anticancer efficacy of Cur-B against the prostate cancer PC3 cell line. During the present investigation, we tried to systematically elucidate the anticancer efficacy of Cur-B against androgen-independent prostate cancer PC3 cells. Our preliminary observations indicated that Cur-B exerted substantial cytotoxic effects by reducing the cellular viability of PC3 cells, which, in turn, was directly proportional to the concentration of Cur-B. The cytotoxic effects of Cur-B on PC-3 cells were further validated by observations during morphological evaluation of PC-3 cells. During morphological assessment, it was evident that exposure to Cur-B instigated the rupturing of PC-3 cell membranes, which was concomitantly followed by their detachment from the plate.

For maintaining the homeostatic conditions within any multicellular organism, regulated apoptosis is a prerequisite. Deregulated apoptotic pathways play a critical role in initiating various carcinomas. Intriguingly, instigation of apoptosis in tumors has now become a well-accepted strategy for clinical management of different carcinomas [19,20,24,25]. Several reports have previously substantiated that apoptosis instigation is concomitantly followed by disrupted nuclear and cytoplasmic morphology, resulting in cell apoptosis [21,22,26,27]. The observations reported herewith were indicative of Cur-B-instigated apoptosis in PC-3 cells by initiating abruption within nuclear morphology, especially fragmentation and condensation as seen in the DAPI- and Hoechst-33342-stained fluorescent photomicrographs. Evasion of apoptotic pathways leads to proliferation of cancer cells, which itself is a prerequisite for tumor progression [23,28]. It is well-accepted that natural products derived from plants hold the intrinsic potential of instigating apoptosis, which subsequently results in restraining the development of various carcinomas [24,29]. The efficacy of Cur-B in initiating apoptosis within prostate cancer PC-3 cells was also reaffirmed through AO/EtBr dual staining. The photomicrographs explicitly indicated that Cur-B treatment significantly increased EtBr-mediated orange intensity, which is characteristically observed in apoptotic cells. Such an observation was further validated by diffused green fluorescence mediated by acridine orange, depicting the loss in viability of Cur-B-exposed PC-3 cells [25,30].

Among several other members of cysteine proteases, caspases have been established as foremost mediators of apoptosis. Caspases, namely caspase-8 and caspase-9, are functionally activated during activation of intrinsic and extrinsic apoptotic pathways, respectively [26,31]. Intriguingly, the downstream activation effects of both caspase-9 and -8 are mediated by capsase-3, which is specifically reported to be responsible for catalyzing proteolytic cleavage of several important cellular proteins [27,32]. The activation of stated caspases post-Cur-B exposure explicitly indicated that Cur-B was a competent in activating apoptosis through intrinsic as well as extrinsic pathways in human androgen-independent prostate cancer PC-3 cells. Importantly, it was also observed that caspase-inhibitor-pre-treated prostate cancer PC-3 cells showed significant amelioration of Cur-B-mediated cytotoxicity. Thus, it was affirmative to conclude that caspases played a crucial role in Cur-B-instigated apoptosis within PC-3 cells. Contrastingly, the caspase inhibitors failed to completely ameliorate Cur-B-mediated toxicity on PC-3 cells, which further indicates that Cur-B exposure may have activated some other caspase-independent apoptosis pathways in PC-3 cells. Therefore, it may be appended that Cur-B-instigated apoptosis in PC-3 cells may have been a result of activated caspase-dependent and/or independent apoptosis pathways.

Being the foremost site of bioenergetics and metabolism, mitochondria are considered to be important cell organelles for evaluating key metabolic situations, including cellular death. Dissipation of ΔΨm serves as an initial trigger for activating apoptosis in cells [28,33]. The observations reported here also indicated that Cur-B succeeded in dissipating ΔΨm directly depending upon Cur-B concentration. This altered ΔΨm could have initiated signal transduction through the mitochondria, resulting in the onset of apoptosis. Another important outcome of dissipated ΔΨm is the concomitant opening of channels governing the mitochondrial permeability, which subsequently results in cytosolic migration of pro-apoptotic proteins, namely, cytochrome-c. Thus, Cur-B mediated apoptosis within treated androgen-independent human prostate cancer PC-3 cells by dissipation of ΔΨm, which has been previously linked to caspase-3 activation [29,34]. The generation of highly unstable singlets during imbalances in metabolic processes often results in generation of oxidative stress, which, subsequently, is further augmented by its insufficient neutralization. ROS are important biomarker of such oxidative stress and are highly unstable. Increased generation of ROS is correlated either directly and/or indirectly with mutilated DNA/RNA, lipids and peptides, all of which acts as instigators for apoptosis. Furthermore, dissipation of ΔΨm has also been reported as an immediate effect of increased ROS within cells, which also inevitably leads to release of pro-apoptotic factors within the cytosol [30,35]. Therefore, we further explored the plausible connection between dissipated ΔΨm and ROS in PC-3 cells after Cur-B treatment. The fluorescent photomicrographs made it evident that Cur-B also instigated the production of ROS in a dose-dependent manner. The increase in DCF-DA-mediated fluorescence intensity indicated the involvement of mitochondria-dependent apoptosis. The observation that Cur-B exposure resulted in production of intracellular ROS in PC-3 cells was further established by the use of a potent ROS inhibitor, namely, NAC. The pre-treatment of NAC considerably ameliorated Cur-B-mediated cytotoxic effects, which were quite prominent in the absence of NAC. Thus, it became clear that increased production of ROS after Cur-B treatment may be considered an important instigator of apoptosis.

Progression of cells through various phases of the cell cycle is an important cellular and molecular event during the lifespan of nearly every mammalian cell. The progression of cells through key phases of the cell cycle is primarily regulated by a family of proteins referred to as cyclins, cyclin dependent kinases (CDKs) and inhibitors of CDKs. The findings of PCR analysis showed that Cur-B exposure succeeded in decreasing cyclinD1 and CDK4 mRNA expression levels. Importantly, previous reports have established that molecular interaction of cyclinD1 with CDK4 serves as an important checkpoint for cell cycle arrest during the G1/S phase [31,36]. Thus, it may be concluded that due to the decrease in cyclinD1 and CDK4 expression, Cur-B would have also resulted in limiting the progression of PC-3 cells at the G0/G1 phase, further explaining the anticancer efficacy of Cur-B against PC-3 cells. Contrastingly, CDK inhibitors or CDKIs such as p21Cip1 and p27Kip1 have also been reported for their regulatory role in restraining the entry of cells in the S phase of the cell cycle by impeding the kinase activity of complexes formed between cyclins and CDKs [32,37]. We also found that p21Cip1mRNA levels were significantly increased, thereby subsequently affirming the notion that Cur-B would have also resulted in limiting the progression of PC-3 cells at the G0/G1 phase. Extensive research over the past several years has made it evident that Bcl-2 protein family members play a considerable role in regulating apoptosis. Such an indispensable role is tightly fine-tuned by Bax, Bad (pro-apoptotic) and Bcl-2 (anti-apoptotic) proteins [33,38]. The observations stated herewith indicated that Cur-B exposure elevated the expression levels of Bax and Bad, which was concomitantly followed by deflated expression of Bcl2. This observation can further be regarded as a testimony that Cur-B exposure led to a considerable increase in mRNA expression of pro-apoptotic genes, followed by a reduction of the levels of anti-apoptotic genes.

As stated previously, STATs play important biological functions involving immune response, cell growth and proliferation. Importantly, they are also associated with the onset and progression of PCa along with various other carcinomas. Despite their structural similarities, members of the STAT family exert independent actions in PCa. STAT1, STAT3 and STAT5 are implicated with resistance to therapy, whereas STAT1 and STAT3 mediate resistance to the standard chemotherapeutic docetaxel. Intriguingly, STAT3 and STAT5 play an important role in resistance during anti-androgen therapy [34,39]. Therefore, we tested our hypothesis that Cur-B exerts anticancer effects against PC-3 by modulating the JAK1/STAT1 signaling pathway. The qPCR analysis supported our hypothesis, since it showed that expression of JAK1 and STAT1 was considerably reduced after exposure of PC-3 cells to Cur-B. However, Western blotting and animal studies are needed to confirm these experimental findings in the future.

## 4. Materials and Methods

### 4.1. Materials

Cucurbitacin-B, Hoechst 33342, capase-8, -9, and -3 inhibitors (ZIETD-FMK (caspase-8), Z-LEHD-FMK (caspase-9), and Z-DEVD-FMK (caspase-3)) and 2,7-dichlorodihydrofluorescein diacetate (DCFH-DA) were commercially obtained from Sigma-Aldrich (St. Louis, MI, USA). Caspase-8, -9 and -3 activity kits were obtained from BioVision, Milpitas, CA, USA. All Ham’s F12K, fetal bovine serum and antibiotic–antimycotic solutions used for culturing PC-3 cells were obtained from Gibco (ThermoFischer Scientific, Waltham, MA, USA). MTT dye, NAC or N-Acetyl cysteine and the total RNA purification kit were procured from HiMedia Labs, Mumbai, India. The cDNA synthesis and qPCR kit (Verso cDNA synthesis) and the DyNAmoColorFlash SYBR Green kit were procured from ThermoFischer Scientific, USA.

### 4.2. Cell Culture

Androgen-independent human prostate cancer PC-3 cells were commercially procured from the cell repository of the National Center for Cell Sciences, Pune, India. The cells were kept in a standard cell culture environment constituting 5% CO_2_ at 37 °C. Ham’s F-12K media complemented with 10% FBS and 1% antibiotic–antimycotic solution was used as media during the entire study protocol.

### 4.3. Methods

#### 4.3.1. Evaluation of Cell Viability Post Cur-B Exposure through MTT Assay

To validate the cytotoxic potential of Cur-B at varying concentrations on androgen-independent prostate cancer PC3 cells, MTT assay was undertaken with subtle modifications [12,35]. A total of 5 × 10^3^ cells/well were exposed to Cur-B at 5, 10, 15, 20 and 25 μM concentrations for 24 h and were incubated in a humidified atmosphere. Subsequently, media of every well was replaced with MTT dye (5 mg/mL; 10 μL) and left for another 4 h for incubation. Formazan crystals were then dissolved using 100 μL of tissue-culture-grade DMSO. Absorbances of Cur-B treated and untreated wells were recorded at 570 nm through spectrophotometer, and cell viability was statistically predicted as cellular viability percentage (%) in comparison with control PC-3 cells.

#### 4.3.2. Morphological Alterations within Cur-B-Treated Prostate Cancer Cells

Cur-B-exposed prostate cancer cells were also examined for alterations within their morphology. Post-overnight incubation, nearly 5 × 10^3^ cells/well were subjected to Cur-B treatment (5–25 μM) and further incubated for 24 h. Ultimately, morphological characteristics of prostate cancer PC3 cells were observed in bright light using a FLoid imaging station, Themo-Scientific, Waltham, MA, USA.

#### 4.3.3. Assessment of Nuclear Condensation

Apoptotic effects in Cur-B-pre-exposed PC3 cells were elucidated qualitatively through DAPI as earlier described [13,36]. PC3 cells were transferred to a 96-well plate, with each well accommodating approximately 6 × 10^3^ cells, and incubated for adherence. Cells were subsequently exposed to varying concentrations (5–25 μM) of Cur-B for 24 h. Post-incubation, the media were decanted, and cells were washed (1X chilled PBS). The treated and control cells were then fixed through chilled methanol (10 min). In the end, cells were permeabilized (3% paraformaldehyde and 0.25% Triton X-100) and stained with DAPI. The cells were visualized at an excitation:emission ratio of 390/40 nm: 446/33 nm through a FLoid imaging station, Thermo-Scientific, USA.

#### 4.3.4. Assessment of Cur-B-Instigated Apoptosis

Instigation of apoptosis within PC-3 cells exposed to Cur-B at stated concentrations was assessed by acridine orange (AO) and ethidium bromide (EtBr) as earlier reported [14,37]. Approximately 5 × 10^5^ PC-3 cells/well were incubated overnight for adherence in a 96-well plate. Thereafter, the cells were treated with 5–25 µM concentrations of Cur-B and incubated for 24 h. Subsequently, the cells were pelleted (1500 rpm, 4 °C for 2 min). The pellet was mixed gently and incubated briefly for around 15 min with 100 µL each of AO and EtBr. In the end, the cells were seen under green and red filters of a fluorescence microscope (FLoid imaging station, Invitrogen, Waltham, MA, USA). The live and dead cells were quantified using ImageJ software (NIH, Bethesda, MD, USA).

#### 4.3.5. Evaluation of Different Caspases Activity

Commercially available caspase-8, -9, and -3 colorimetric kits were utilized to demonstrate the activities of different caspase in human-derived PC3 cells as per the manufacturer’s instructions. Post Cur-B exposure (at the above-stated concentrations), 3 × 10^6^ prostate cancer cells were lysed by 50 μL lysis buffer (chilled; 10 min incubation on ice). The resulting suspension was then centrifuged (10,000 rpm for 1 min at 4 °C), and the supernatants were accumulated. A total of 50 μL lysate was thereafter added in each well of a 96-well plate with the addition of 50 μL reaction buffer. Four mM substrate (DEVD-pNA) was also supplemented in every well and briefly incubated (10 min). Finally, absorbance of each well was recorded at 405 nm using a microplate reader (Bio-Rad, Hercules, CA, USA). Percentage increase of the activities of different caspases was calculated by comparing the changes with the level of untreated PC3 control cells.

#### 4.3.6. Assessment of Caspase Inhibitor Pretreatment on Cur-B Exposure

Cur-B modulated cytotoxicity on PC3 cells was further delineated through treatment with caspase-8, -9, and -3 inhibitors. PC3 cells were initially treated for 2 h with ZIETD-FMK, Z-LEHD-FMK, and Z-DEVD-FMK (50 μM; caspase-8, -9, and -3 inhibitors, respectively). Subsequently, the cells were re-exposed to varying stated concentrations of Cur-B and incubated for an additional 24 h. Eventually, the viability of Cur-B-treated PC3 cells was evaluated as cell viability percentage following the procedure described above in Section 4.3.1.

#### 4.3.7. Qualitative Assessment of Mitochondrial Membrane Potential (ΔΨm)

Cur-B-mediated effects on ΔΨm modulation within PC3 cells was qualitatively evaluated using Rhodamine (Rh)-123 stain as previously stated [15,38]. Concisely, 5 × 103 PC3 cells were seeded in each well of a 96-well plate and were allowed to incubate overnight in ambient culture conditions. Subsequently, these were exposed to varying stated concentrations of Cur-B for 24 h. Post Cur-B exposure, the cells were re-treated with 5 mg/mL of Rh-123 in the dark for 30 min. Green fluorescent photomicrographs of PC3 were recorded using a FLoid imaging station (Thermo-Scientific, USA).

#### 4.3.8. Evaluation of Cur-B-Instigated ROS

Instigation of ROS post Cur-B exposure within PC3 cells was qualitatively assessed through DCFH-DA stain as per the procedure described earlier [16,39]. Precisely, 5 × 103 PC3 cells/well were incubated overnight in a 96-well plate to allow adherence under optimum culture conditions. Thereafter, cells were treated with Cur-B (at stated concentrations) and incubated for an additional 12 h. Media from each well was decanted, and cells were re-treated with 10 μM DCFH-DA (30 min incubation at RT in dark). At the concluding step, the cells were washed using PBS prior to imaging at a wavelength ratio of excitation: 482/18 nm–emission: 532/59 nm through the green channel of a FLoid imaging station, Thermo-Scientific, USA.

#### 4.3.9. Real-Time qPCR Analysis

Approximately 1 × 10^6^ cells/well/mL PC3 cells were allowed to adhere overnight under optimum conditions. Post-adherence, these cells were subjected to varying Cur-B concentrations for 24 h before total RNA extraction using commercially available kits. The cDNA was synthesized from the extracted RNA, and qRT-PCR analysis was further undertaken using a SYBR Green qPCR Kit as per the standard procedure to evaluate alterations within the Bax, Bad, Bcl2, CDK4, p21^Cipl^, cyclinD1, JAK1 and STAT1 genes. A comparative CT method was used to analyze levels of gene expression variations with constitutively expressed GAPDH control genes and by setting the standard for the control group to 1. The primers used were designed using the NCBI primer designing tool as shown in Table 1.

#### 4.3.10. Statistical Evaluation

Data represent mean ± SEM of three independent experiments, each performed in triplicate. Significance among different dosage groups was determined by GraphPad Prism (Ver. 5) using one-way ANOVA followed by Dunnett post-hoc test and two-tailed test.

## 5. Conclusions

The results reported in the present investigation elucidated that Cur-B-instigated downregulation of JAK/STAT signaling played a critical role in upregulating the expression of pro-apoptotic Bax and Bad proteins with concomitant alleviation of the anti-apoptotic Bcl-2 gene. Furthermore, Cur-B also elevated the mRNA expression of genes involved in increasing the arrest of the cell cycle in the G0/G1 phase, followed by activation of both extrinsic and intrinsic apoptotic pathways. Our report thus provides a mechanistic insight into the anticancer effects of Cur-B, thereby indicating the plausible chemopreventive role of Cur-B against androgen-independent human prostate cancer PC-3 cells.

## Figures and Tables

**Table 1:** List of forward and reverse sequences of primers used during qRT-PCR evaluation.

Gene Name	Forward Sequence	Reverse Sequence
GAPDH	CGACCACTTTGTCAAGCTCA	CCCCTCTTCAAGGGGTCTAC
Bax	GCTGGACATTGGACTTCCTC	CTCAGCCCATCTTCTTCCAG
Bad	CCTCAGGCCTATGCAAAAAG	AAACCCAAAACTTCCGATGG
Bcl2	ATTGGGAAGTTTCAAATCAGC	TGCATTCTTGGACGAGGG
cyclinD1	CTTCCTCTCCAAAATGCCAG	AGAGATGGAAGGGGGAAAGA
CDK4	CCTGGCCAGAATCTACAGCTA	ACATCTCGAGGCCAGTCATC
p21^Cip1^	TGTCCGTCAGAACCCATG	GTGGGAAGGTAGAGCTTGG
JAK1	ATCCTTCGCACAGACAACATC	GCATTCCTGAGCCTTCTTGG
STAT1	ATGGCAGTCTGGCGGCTGAATT	CCAAACCAGGCTGGCACAATTG

## Data Availability

Data is contained within the article.
